# BioOne: a national-scale platform for integrated discovery and utilization of diverse biological resources in South Korea

**DOI:** 10.1186/s44342-026-00070-x

**Published:** 2026-04-08

**Authors:** Tae-Ho Kang, KyoungSoo Ha, WonHyun Kyung, Yong-Jin Jeon, Geun-Hyoung Jo, KangMin Park, KwangHee Lee, Tae-Eun Jin

**Affiliations:** Korea Bioinformation Center (KOBIC), Korea Research Institute of Bioscience & Biotechnology (KRIBB), Daejeon, Republic of Korea

**Keywords:** BioOne, Biological resources, Metadata integration, Interoperability, Biological database

## Abstract

The rapid growth of biological resources and associated research outputs has increased the complexity of resource discovery, access, and reuse across heterogeneous repositories. However, fragmented metadata schemas, limited interoperability, and siloed access mechanisms continue to hinder the integrated exploration of biological resources and their associated knowledge. Herein, we present BioOne (Biological resources One-Stop service platform), a unified informatics framework designed to address the fragmentation of national biological resources by integrating heterogeneous metadata from 14 distinct clusters and establishing a seamless resource-to-knowledge pipeline to enhance the discoverability and practical utilization of biological assets.

BioOne is a national-scale web-based discovery platform that harmonizes biological resource metadata across 14 domain-specific biological resource clusters in Korea and systematically links these resources with external knowledge objects, including research papers, patents, biological datasets, and disease–drug–target information, through a unified discovery interface. To achieve this, we adopted a standard-aligned metadata integration framework, interoperable identifier mapping, and a modular system architecture to support scalable indexing, cross-domain search, and association-based navigation.

By extending conventional catalog-based biological resource databases with an integrated discovery and access layer connecting distributed biorepositories to evidence-oriented knowledge resources, BioOne provides an informatics infrastructure for data-driven discovery, translational research, and coordinated utilization of biological resources at the national scale. The BioOne also offers a transferable implementation model for the large-scale integration of distributed biological resource systems.

**Supplementary Information:**

The online version contains supplementary material available at 10.1186/s44342-026-00070-x.

## Background

Biological resources and their associated experimental and analytical data are foundational assets for modern life science research [1, 2]. Advances in high-throughput technologies have substantially increased the volume and diversity of biological data, intensifying the need for systematic management, integration, and use of biological resources [3–5]. These resources underpin diverse research activities, including functional genomics, disease mechanism studies, drug discovery, and agricultural biotechnology [6–8].

Despite the proliferation of biological resource centers and repositories worldwide, biological resource information remains fragmented across independent platforms with heterogeneous metadata structures and access policies, limiting cross-domain discovery, hampering reproducibility, and increasing the effort required to identify and evaluate suitable resources [9, 10]. Researchers often need to navigate multiple portals to supplement inconsistent metadata and manually integrate contextual evidence from research papers, patents, and datasets [11, 12]. The NCBI (National Center for Biotechnology Information) in the USA [13], ELIXIR (European life-sciences infrastructure for biological information) infrastructure in Europe [14], and NBDC (National Bioscience Database Center) in Japan [15] exemplify global efforts to integrate biological resources and data, all emphasizing standardized metadata and unified access.

To address these challenges, we developed BioOne (Biological resources One-Stop service platform), a national-scale platform that integrates distributed biological resource metadata and associated research outputs into a unified discovery environment [4, 9, 16–19]. By harmonizing heterogeneous metadata and associating biological resources with relevant scientific and technological evidence, BioOne facilitates efficient resource discovery and knowledge-driven utilization across research domains.

## Construction and content

In accordance with the Third National Strategy for the Management and Utilization of Biological Research Resources of Korea, national biological resources are organized into 14 major biological resource clusters to enable systematic management and domain-specific utilization [17, 19]. As described in Supplementary Table S1, these clusters include animal models, cell lines, chemical compounds, fishery resources, human brain resources, human derived resources, livestock, marine resources, microbes, natural products, pathogens, seeds, stem cells, wildlife resources [19]. These 14 clusters represent a strategic reorganization of national biological assets into specialized domains, each governed by unique curation standards and quality controls tailored to specific resource types. Although the pathogen cluster prioritizes bio-safety and epidemiological data, the seed cluster focuses on agricultural traits and genetic diversity. BioOne bridges these distinct domains by harmonizing heterogeneous metadata into a standardized discovery layer. This integration enables cross-disciplinary research, which was previously hindered by siloed management, by systematically linking biological resources with associated knowledge objects, including research papers, patents, and biological datasets.

BioOne integrates metadata and associated research outputs across all 14 clusters, providing a unified discovery environment while preserving the resource information, curation practices, and distribution workflows unique to each cluster. BioOne is a centralized, web-based platform designed to integrate heterogeneous biological resource information through standardized interfaces in accordance with the Korean Standard for Biological Resource Data Integration, as described in Supplementary data 1. The platform adopts a multimodal data collection strategy to construct a comprehensive database encompassing biological resource metadata, scientific research papers, patents, biological datasets, disease information, and drug data [20–23].

**Fig. 1:**
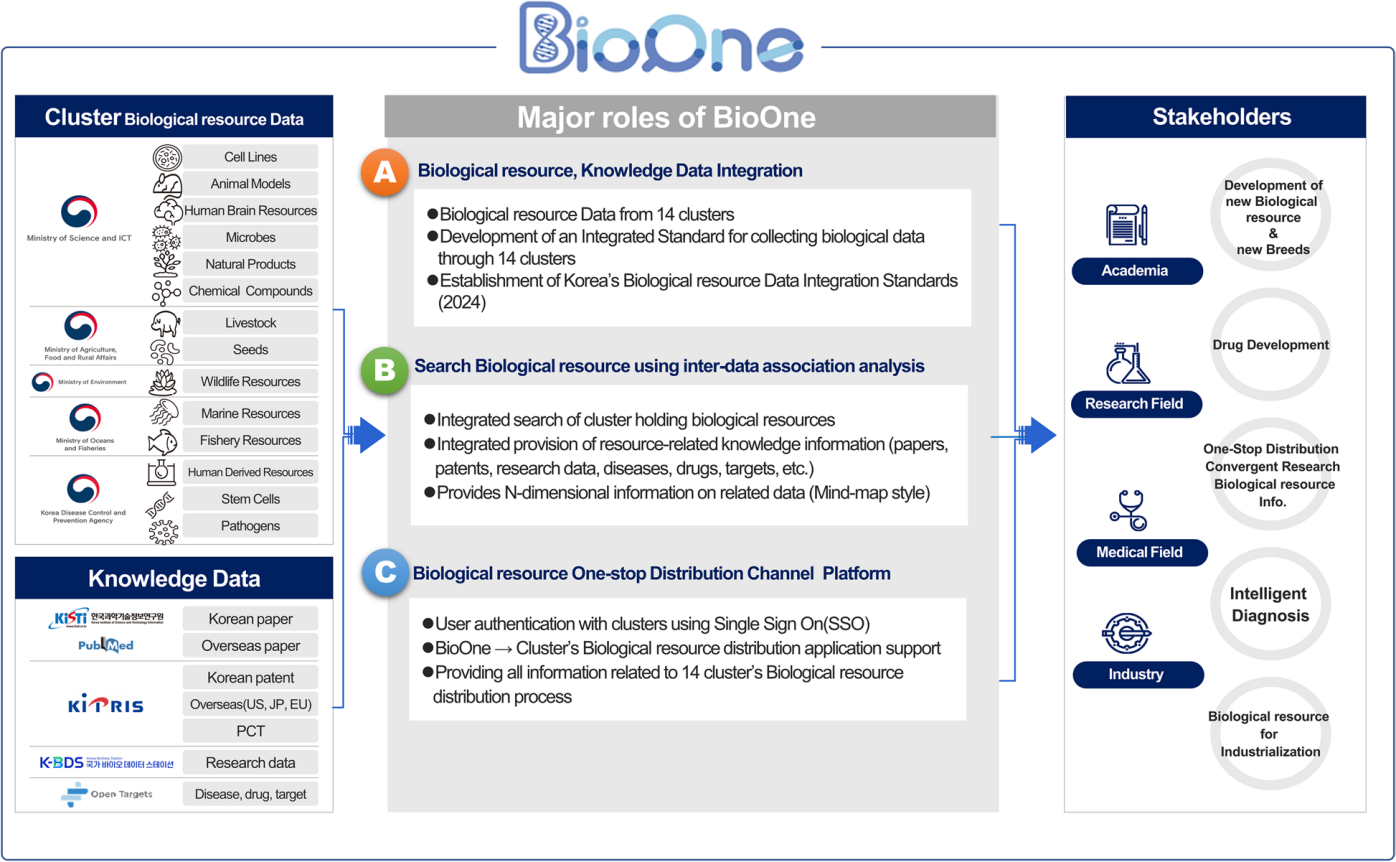
Conceptual framework and core functionalities of BioOne. BioOne integrates fragmented biological resource metadata from 14 national clusters into a standardized framework. **A** Data integration: heterogeneous metadata were harvested and normalized using automated pipelines and the Korea Biological Resource Data Integration Standard. **B** Knowledge enrichment: resources were systematically linked to external knowledge objects, including publications, patents, and disease–drug–target datasets. **C** Discovery services: the platform provides a unified search interface (Elasticsearch-based), category-specific navigation, and association-based discovery tools. This architecture enables researchers to explore resources within broader functional and scientific contexts through a single entry point

As illustrated in Fig. 1, the conceptual framework and core functionalities of BioOne include a biological resource data integration system, an integrated search environment, semantic enrichment, and an integrated access-and-distribution workflow interface. BioOne provides a robust integrated search engine spanning all indexed data types, including biological resources, research papers, and patents, allowing researchers to explore cross-domain connections using a single query [9, 24].

Simultaneously, the platform supports cluster-specific navigation, enabling experts to perform in-depth searches within a particular biological domain while maintaining the unique metadata context of each cluster [20, 25]. Elasticsearch, which facilitates real-time indexing and complex query processing, powers the search capabilities of the platform [25].

### System architecture

**Fig. 2:**
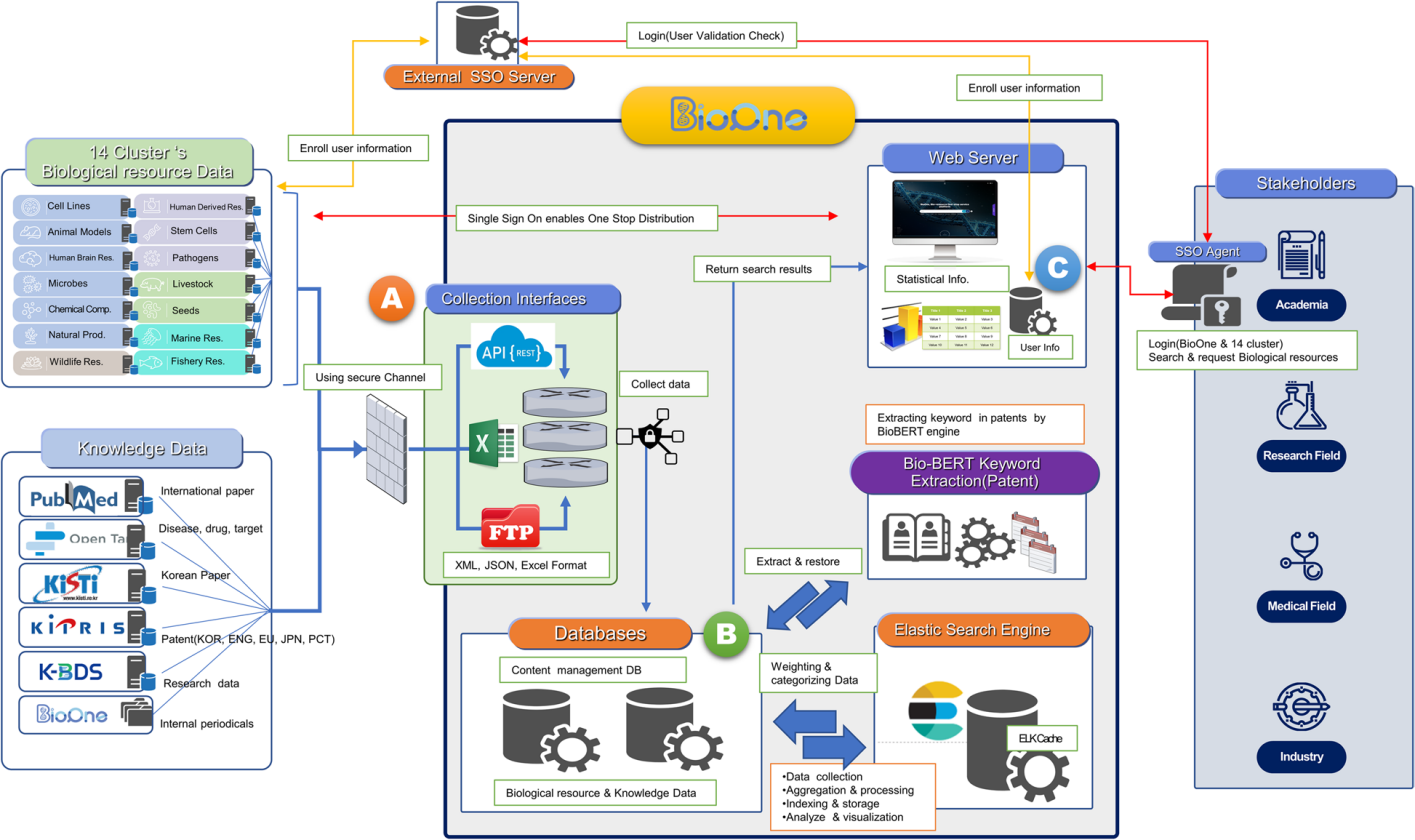
Technical system architecture of BioOne. This architecture is designed with a logical flow, moving from raw data acquisition to refined user services. It comprises three functional layers. **A** Data acquisition layer: this layer collects standardized metadata for biological resources provided by 14 national resource clusters. It collates domestic and international knowledge data, including academic papers, patents, research data, drugs, targets, and genetic information. **B** Standardization and integration layer: this layer standardizes heterogeneous data into a unified schema. It performs search indexing using Elasticsearch to improve the search efficiency. **C** Service layer: this layer provides a user interface for searching and establishing knowledge connection. The arrows in the diagram represent the data flow, beginning with raw data collection and leading to an integrated knowledge output

BioOne is a multilayered informatics framework designed to bridge the gap between fragmented biological resource repositories. As shown in Fig. 2, the system architecture consists of three primary functional layers. First, the Data Acquisition Layer harvests heterogeneous metadata from 14 national resource clusters via RESTful (Representational State Transfer) application programming interfaces (APIs) [26]. Second, the Integration and Processing Layer transforms raw data into a unified schema, ensuring semantic interoperability. Finally, the Service Layer automatically links integrated resources with external knowledge objects, such as PubMed, GenBank, and patent databases. This systematic workflow allows users to navigate from a biological resource to its functional genomic data and associated research outcomes within a single interface [6, 27, 28].

Additional data sources, including biomedical research papers and disease–target databases, were integrated through API-based pipelines and pre-processed using Logstash before indexing in Elasticsearch [26, 27, 29]. Patent data were extracted from XML (eXtensible Markup Language)-based sources and transformed into structured JSON (JavaScript Object Notation) formats using Python-based pipelines, enabling the integration of complex intellectual property metadata.

Direct database-to-database synchronization via validated database views was used to ensure data consistency and prevent duplication [30]. This architecture minimizes manual intervention and enhances the long-term maintainability.

### Data and knowledge integration

BioOne functions as a centralized gateway that integrates multiple specialized biological resource portals and external knowledge bases. Core biological resource metadata—including taxonomy, origin, storage conditions, availability, and application procedures—are harmonized to ensure consistent representation across resource types [5, 9, 18, 31].

Table 1 summarizes the external data sources integrated with BioOne to support an integrated discovery environment. The sources are grouped into four functional domains: biological resource information, literature and patents, drug and disease informatics, and research results.

**Table 1:** Categorization and functional roles of external data sources integrated into BioOne

Category	Data source	URLs	Information provided	Records
Biological resource information	Specialized biological resource portal	Cluster-specific portals for 14 national biological resource domains	Each cluster provides access to its own biological resources through a dedicated portal	1,167,776 (2025.12.22)
Literature and patents	PubMed (International Papers)	https://pubmed.ncbi.nlm.nih.gov	International biomedical literature access through NCBI APIs	39,827,329 (2025.12.22)
	KISTI (Korean Papers)	https://scienceon.kisti.re.kr	Korean bio-related scientific publications	290,053 (2025.12.22)
	KIPRIS	https://www.kipris.or.kr	Integration with the patent data	2,122,338 (2025.12.22)
Drug and disease	Open Target Platform	https://platform.opentargets.org	Integration with disease, target, drug data	136,376 (2025.12.22)
Research results	K-BDS	https://kbds.re.kr	All data generated and used through Korean Bio R&D	156,982 (2025.12.22)

As described in Table 1, the platform integrates international and Korean research paper databases, patents, and biological data, enabling users to explore biological resources within a broader scientific and technological context. BioOne supports the comprehensive evaluation of resource relevance, novelty, and application potential by linking biological resources to research papers, patents, and research datasets [9, 24].

### Search, semantic enrichment, and visualization

BioOne leverages the Elasticsearch–Logstash–Kibana (ELK) stack to support scalable indexing, real-time retrieval, and interactive visualization [16]. Advanced text analysis techniques, including language-specific tokenizers and synonym dictionaries, improve the search accuracy across multilingual and heterogeneous datasets [25]. Text-based retrieval is optimized using analyzer-level configurations rather than relevance-score tuning. BioOne applies the Nori tokenizer for Korean-language processing [26], an N-gram analyzer for partial matching, and a curated synonym dictionary for term normalization across data types. The search results and associated relationship-based visualizations were ranked using the default BM (Best Matching) 25 relevance scoring of Elasticsearch.

BioOne applies BioBERT (Bidirectional Encoder Representations from Transformers for Biomedical Text Mining), a biomedical domain–specific language model, to extract representative keywords from patent claims and related texts to address the limited keyword metadata typically associated with patent documents [32]. The extracted terms were indexed as supplementary metadata, enabling improved discoverability of patent records and supporting context-aware keyword recommendations for related publications and research datasets. Semantic enrichment is used to improve recommendations and keyword expansion services across research papers and datasets [24, 32, 33].

Search results are presented through tabular views and interactive radial visualization interfaces, enabling the intuitive exploration of relationships among biological resources, research papers, patents, and datasets [25, 34].

**Fig. 3:**
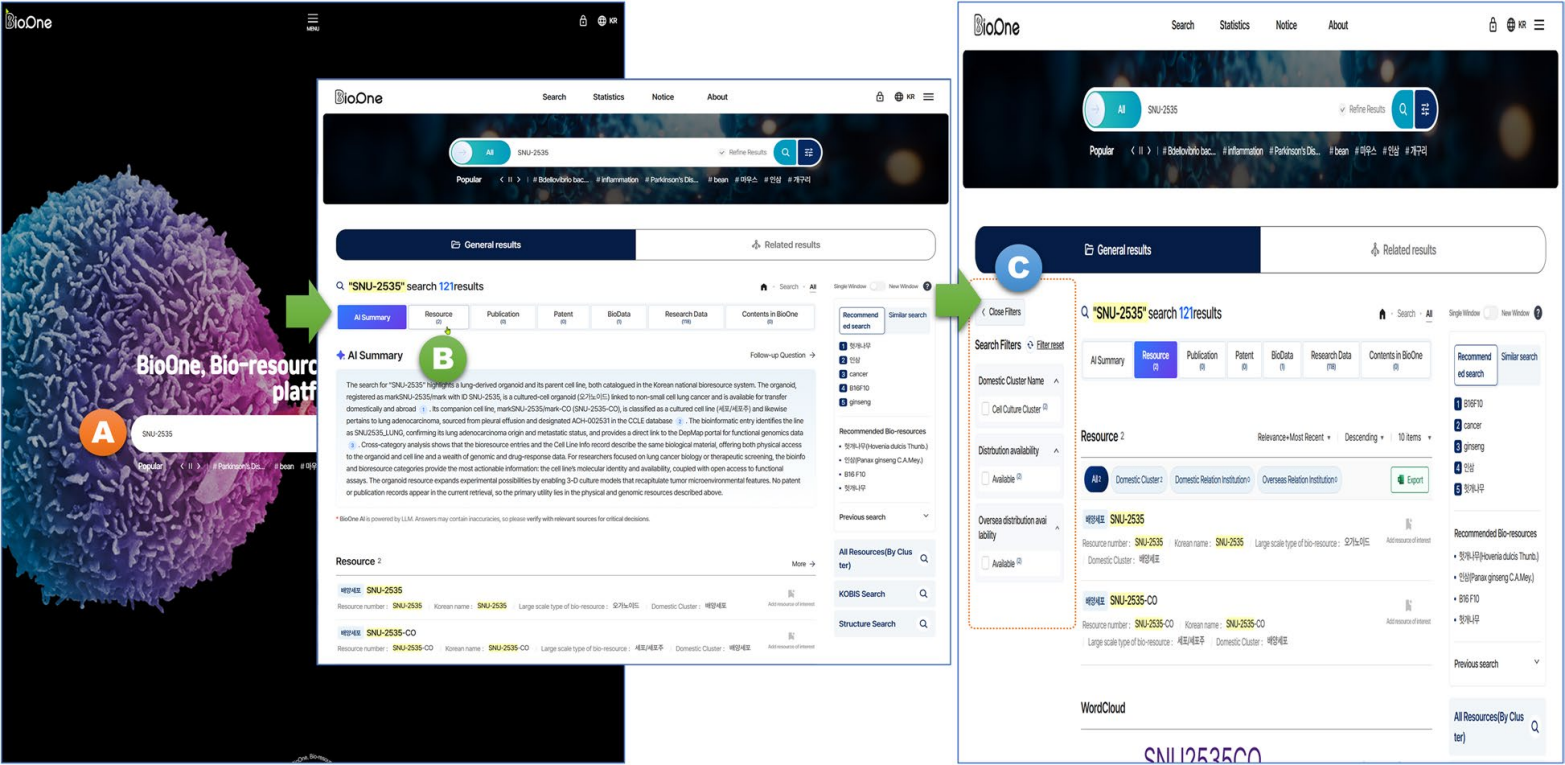
Stepwise workflow of the BioOne discovery interface. **A** Step 1: the user enters a search query (e.g., “SNU-2535”) within the BioOne search panel to retrieve relevant biological resource records. **B** Step 2: on the search results screen, users can select the resource menu to activate the detailed search panel (left panel), and then use it to limit the search results for biological resources to the cluster or specific knowledge data of their choice. **C** Step 3: BioOne displays a consolidated results page integrating biological resource records with linked knowledge objects, including related publications, patents, and biological datasets. The side menu provides various filtering options to refine the results by resource type and source, enabling users to identify and utilize relevant resources more effectively

Figure 3 summarizes the major roles of BioOne in the national biological resource ecosystem to clarify the scope and positioning of the platform beyond its technical architecture. In addition to aggregating standardized metadata across 14 cluster-specific portals, BioOne links biological resources with evidence-oriented knowledge objects, including research papers, patents, and disease–drug–target information, to support discovery-driven utilization. This conceptual view highlights how integrated search and association-based exploration in BioOne facilitates stakeholder workflows from resource identification to integrated access and distribution. A comprehensive user manual detailing the search functionalities and association-based navigation is available through the BioOne website to support accessibility and ease of use for diverse researchers [https://www.bioone.re.kr/help/en/index.html].

### Performance and security

BioOne uses a single sign-on (SSO) authentication framework to provide seamless access across integrated biological resource clusters [35, 36]. Centralized identity management ensures secure and uniform authentication through a trusted identity provider (IdP), simplifying security policy enforcement, user provisioning, and access monitoring [14, 37]. This architecture is intended to improve system security, operational manageability, and user convenience.

The implementation of SSO reduces administrative overhead by eliminating the need for individual authentication systems at each biological resource cluster, thereby enabling centralized and consistent user access management. Consequently, IT support costs and login-related inquiries were minimized [37]. SSO systems rely on standard authentication protocols, such as Security Assertion Markup Language or OpenID Connect, allowing secure token exchanges between BioOne and the central IdP [36, 37].

BioOne streamlines biological resource procurement through integrated authentication and one-stop access to specialized biological resource distribution portals. This integration reduces administrative overhead and supports a more efficient biological resource acquisition process through centralized identity management. [37, 38].

As described in Supplementary data 2, benchmark testing demonstrated stable performance under concurrent user access within an on-premises, secure research network [30]. The system operation complies with national information security policies, with future plans for secure external deployment to improve global accessibility [17, 38].

## Utility and discussion

BioOne differs from general-purpose information retrieval tools in that it supports validated resource discovery together with access pathways. For example, when a user searches for “SNU-2535,” BioOne can present the resource record together with linked research outputs, the corresponding repository or cluster information, and the access route for distribution through the relevant center. By contrast, general-purpose language models or web search tools may summarize textual information related to “SNU-2535,” but they do not natively provide validated provider information or integrated distribution procedures within the same interface. In this sense, BioOne supports a resource-oriented workflow that extends beyond information retrieval to practical resource identification and acquisition.

BioOne addresses persistent challenges in biological resource discovery through an integrated platform based on metadata harmonization and an interoperable system design. Unlike conventional biological resource portals that operate as isolated, resource-type–specific repositories, BioOne functions as a cross-domain integration layer that connects biological resources with relevant scientific, technological, and biomedical evidence [9, 29]. The platform supports exploratory discovery workflows that extend beyond simple catalog-based access by situating biological resources within a broader data-driven research environment.

A key contribution of BioOne is its standardized metadata integration framework based on the Korean Standard for Biological Resources Data Integration described in Supplementary data 1. The platform reduces structural inconsistencies that impede cross-platform discovery, a challenge common in multi-institution and multidisciplinary data integration settings, by harmonizing heterogeneous metadata across biological resource clusters and aligning them with internationally recognized principles [5, 24, 28]. BioOne preserves cluster-native identifiers while assigning unified internal identifiers for indexing and cross-linking [5, 9]. This dual-identifier strategy enables seamless integration without disrupting the existing cluster-level distribution and management workflows [9, 28].

From an informatics perspective, BioOne extends beyond conventional catalog-style databases by incorporating semantic enrichment and relationship-driven exploration [24, 32, 33]. The integration of research papers, patents, diseases, and drug information enables users to contextualize biological resources within the existing knowledge landscape [21, 22, 24]. The application of domain-specific language models to under-annotated text sources, particularly patent documents, enhances discoverability by mitigating the limitations of keyword-sparse metadata [32, 33]. Although automated semantic extraction may introduce noise, this approach substantially improves recall in exploratory search scenarios, which are common in early-stage biological resource utilization [24, 32].

Compared with conventional integration approaches, BioOne adopts a standardized metadata harvesting model to support more consistent data integration across diverse biological domains [5]. Unlike link-based systems that primarily provide redirection, mediator/wrapper-based federation models that depend on source-specific query translation, and real-time API-based approaches that may be affected by source-side variability, BioOne relies on pre-integrated and indexed metadata within a unified schema [30]. This design supports more stable query performance, reduces dependency on source-side interface changes, and improves cross-domain consistency in resource discovery.

In relation to platforms such as NCBI (Entrez) and ELIXIR, BioOne has a distinct functional scope in the context of biological resource utilization [14, 20]. Whereas NCBI and ELIXIR primarily support broad life science data access and analysis, BioOne is specifically designed to manage provenance and utilization metadata associated with biological resources. Through a unified discovery interface, BioOne links biological resources with related knowledge objects, including publications, patents, and disease–drug–target information, to support resource-oriented exploration and utilization.

BioOne is complementary to existing Korean biological data infrastructure, particularly the Korea BioData Station (K-BDS). Although the K-BDS focuses on the long-term archiving and sharing of raw biological research data generated through Korean R&D projects, BioOne focuses on the discovery and utilization of biological resources and their associated contextual knowledge [17, 19, 23]. Linking biological resources to datasets archived in K-BDS allows researchers to trace experimental evidence across biological samples and digital data, clarifying functional separation while enabling synergistic interoperability [5, 33].

Currently, full integration has been achieved for a subset of Korean biological resource clusters, and metadata consistency varies across resource types due to differences in curation practices and update frequencies [9]. Semantic enrichment based on automated language models requires continuous validation and refinement to improve precision [32, 33]. Addressing these challenges requires continuous standardization efforts, the incorporation of ontology-driven annotations, and iterative evaluation using curated reference datasets [5, 24].

BioOne functions as an implementation-level infrastructure that translates cluster-based governance into an operational discovery platform by integrating biological resources across 14 nationally designated clusters defined in the Third National Strategy [17, 19]. This design enables cross-cluster exploration and comparative analysis while maintaining cluster-specific management and distribution workflows, thereby supporting both centralized visibility and decentralized stewardship of the biological resources. In this context, BioOne is well positioned to support the Fourth National Strategy for the Management and Utilization of Biological Research Resources by providing a scalable foundation for expanded cluster integration, ELK-based discovery services, and strengthened linkages between biological resources and data-driven research ecosystems.

## Conclusion

BioOne presents a practical implementation of a national-scale biological resource discovery platform that consolidates distributed biological resource metadata and associated research objects into a unified access framework. The platform improves biological resource discoverability and supports a better understanding of relationships among resources by harmonizing heterogeneous metadata, applying interoperable identifier mapping, and linking biological resources with research papers, patents, and biological datasets. This integrated approach reduces fragmentation in research workflows and supports more efficient identification and utilization of biological resources.

Beyond the initial 14 biological resource clusters, BioOne provides an architectural basis for the future integration of additional resource centers. New sites can be incorporated through schema mapping to the BioOne core metadata model, automated harvesting through standardized interfaces, and identifier normalization for cross-resource linking. This modular design provides a scalable framework for expanding into additional domains while maintaining existing search functions, metadata consistency, and association-based discovery services.

In addition to its immediate technical contributions, BioOne establishes an infrastructure model for coordinated biological resource utilization at the national level. The modular architecture, standard-aligned metadata framework, and complementary integration with the K-BDS provide a scalable foundation for long-term sustainability and international interoperability. With the accumulation of biological data and resources in terms of scale and complexity, platforms such as BioOne will be essential for data-driven research, fostering interdisciplinary collaboration, and maximizing the scientific value of biological resources.

## Supplementary Information

Supplementary Material 1: Data 1. Korea Biological Resource Data Integration Standard.

Supplementary Material 2: Data 2. Benchmarking test results of BioOne.

Supplementary Material 3: Table S1. Classification and functional overview of the 14 national biological resource clusters integrated into BioOne.

## Data Availability

BioOne is accessible online, and the list of integrated data sources is provided in Table 1.
