# Supplementary material for: BioOne: a national-scale platform for integrated discovery and utilization of diverse biological resources in South Korea
Source: Genomics Inform. 2026 Apr 8;24:9. doi: 10.1186/s44342-026-00070-x (PMC13130625; doi:10.1186/s44342-026-00070-x)
Supplement: Supplementary file 1 — Supplementary Material 1: Data 1. Korea Biological Resource Data Integration Standard. [file 44342_2026_70_MOESM1_ESM.docx]

**[Supplementary Data 1] Korea Biological Resource Data Integration Standard**

ㅇ Data linkage fields for biological resource

| **Category** | **Attribute** | **Description of attribute** | **Integration** | |
| --- | --- | --- | --- | --- |
|  |  |  | **Required** | **Optional** |
| **Header Info.** | **Institution code** | A unique code that identifies the institution(e.g. KCTC, RIKEN, ATCC, etc) | ○ |  |
| **General**  **Info.** | **Resource ID** | A unique identifier managed by the institution | ○ |  |
|  | **Taxonomic name** | A taxonomic name given to a species of organism, using a system of nomenclature consisting of the names of the species and genus | ○  (Min.  1 attribute) |  |
|  | **IUPAC name** | Name according to IUPAC nomenclature of chemical compounds |  |  |
|  | **Resource name**  (Variety name etc) | Resource names based on the same scientific name(common name) or the concept of genetic diversity, such as cultivar name, system name, local name, or strain name(e.g. Koshihikari, Shine muscat) |  |  |
|  | **Registration date** | Date the resource was registered(8 digit number eg. 19991230) | ○ |  |
|  | **Distribution availability** | Choose one of Y, N, Q(Need to contact) * Indication of availability for 3^rd^ party distribution | ○ |  |
|  | **Export availability** | Choose one of Y, N, P(Need to permission), A(Need to approval), R(Need to Report) | ○ |  |
|  | **Category of resources**(types) | [Table 2] ‘Resource type (form)’ item presented in the Biological resource Type item | ○ |  |
|  | **URL for resource** | Resource URL provided by provider |  | ○ |
|  | **URL for distribution application** | Link to the data provider’s distribution application webpage |  | ○ |
|  | **Image URL** | Resource image URL provided by provider |  | ○ |
|  | **Search keywords** | Additional terms or keywords that can symbolize or describe a resource(Terminology for technology, diseases, etc.). (In case of multiple, specify by separating with ‘ \|\| ’) |  | ○ |
|  | **Origin of resource**  (or Origin of isolation) | The source or origin from which a resource is isolated. |  |  |
|  | **Resource history** | Information on the origin of the resource(if multiple, separate with ‘\|\|’) |  |  |
|  | **Resource ID of other BRCs** | Resource ID used by other BRCs |  |  |
|  | **Chemical structure** | Molecular structure that expresses the atoms and the relationships between the atoms that make up a compound |  |  |
|  | **SMILES notation** | Structure of the compound represented in SMILES format |  |  |
|  | **Predicted property values** | A value that can predict a physical, chemical or biological properties |  |  |
|  | **Chemical name**  (Drug name) | Name of the chemical compound(or drug) |  |  |
|  | **Molecular formular** | Molecular formula of the chemical compound |  |  |
|  | **Target name** | Name of the target of the substance, referring to the molecule or protein within the body that the substance binds to or acts upon to regulate or affect a specific disease or biological process. |  |  |
|  | **Ingredient name** | The name of the component that makes up a substance |  |  |
|  | **Molecular weight** | Molecular weight of a compound |  |  |
|  | **PubChem CID** | PubChem compound identifier(CID) |  |  |
|  | **Country of origin** | The country(or region) that collected or isolated the resource |  |  |
|  | **Family Name** | In biology, the taxonomic English name of a families of resource |  |  |
|  | **Resource classification** | The status of the resource which is expressed in multiple ways, such as rare, extinct, endemic, cultivated, native, and wild relatives |  |  |
|  | **Collector/Isolator** | Affiliation and name of the person(s) who first collected (gathered) the resource from the wild or locally |  |  |
|  | **Publication information of resource** | Title of paper 1((source URL))\|\|Title of paper 2((source URL))\|\|Title of paper3 ((source URL))  (If there are multiple sources, indicate them in double parentheses(( )), and indicate the distinction between papers by separating them with ‘\|\|’) |  |  |
|  | **Clinical diagnosis** | In case of multiple, specify by separating with ‘ \|\| ’ |  |  |
| **Characteristic**  **info.**  **(Focused on Biological resource)** | **Autopsy diagnosis** | In case of multiple, specify by separating with ‘ \|\| ’ |  |  |
|  | **Disease name** | English name of the disease |  | ○ |
|  | **Efficacy**  (Anti-cancer, anti-inflammatory, etc) | [Table 3] ‘Detailed classification’ item presented in the Biological resource efficacy information |  |  |
|  | **Efficacy test status** | Only the implementation status of the efficacy test is indicated (efficacy results are provided through the Efficacy Information Utilization Agreement) |  |  |
|  | **Gene name** | Officially assigned gene name |  |  |
|  | **Mutant type** | Classification of various types of gene mutations |  |  |
|  | **Strain name** | A systematically assigned name for a unique group, individual, entity, or substance within a specific population or set, representing a taxonomic unit below the species level |  |  |
|  | **Human gene info.**  (Association gene) | Human genes associated with specific traits or diseases in genetic studies |  |  |
|  | **Biosafety Level**(BSL) | Biosafety facility level reported in accordance with Article 22 of the Act on Transboundary Movements of Living Modified Organisms |  |  |
|  | **Registrant** | Information of the person who registered the resource (practitioner) |  |  |
| **Tailer**  **Info.** | **Researcher**  (Principal investigator) | Information about the person who studied the resource(researcher or Principal investigator) |  |  |
|  | **Resource source DB** | Reference site or DB Information |  |  |
|  | **Deletion availability** | Indicates whether the resource has been deleted from newly updated materials. | ○ |  |
|  |  |  |  |  |

ㅇ Description of biological resource types

| **NO** | **Resource Type** | **Biological resource description** |
| --- | --- | --- |
| **1** | **Specimen** | In biology, etc., treatment is performed to preserve all or part of a natural object for research or teaching purposes. |
| **2** | **Object** | An inseparable, independent organism with structural and functional features necessary for survival |
| **3** | **Organ** | In multicellular organisms, a structure in which several tissues are combined to form a certain shape, have a certain function, and are morphologically independent. |
| **4** | **Tissue**(including paraffin blocks) | A group of cells that form a living organism and have the same function and form |
| **5** | **Embryos**(including trophoblast) | An organism in the early stages of individual development in the development process of multicellular organisms such as plants and animals. |
| **6** | **Seeds** | Seeds, mushroom spawn, seedlings, spores or vegetative bodies such as leaves, stems and roots used for propagation or cultivation |
| **7** | **Sperm** | In livestock, a fluid secreted from the gonad or other reproductive organs of male animals and containing sperm |
| **8** | **Fertilized egg** | A eukaryotic cell formed by the fertilization of sperm and egg cells of livestock, containing all the genetic information necessary to form a new individual. |
| **9** | **Cell, cell-line** | The functional and structural basic unit of almost all living organisms, a clone of cultured cells that can continue to divide and multiply through cell culture to produce offspring |
| **10** | **Organoids** | Organ-specific cell aggregates created by re-aggregating and recombining cells isolated from stem cells or organ-derived cells using a three-dimensional culture method |
| **11** | **Bacteria** | A representative taxonomic group of prokaryotes, which, along with archaea and eukaryotes, occupies one of the three domains of life. |
| **12** | **Archaea** | A representative taxonomic group of prokaryotes, which, along with bacteria and eukaryotes, occupy one of the three domains of life. |
| **13** | **Fungi** | One of the classifications of eukaryotes, including yeast, mold, and mushrooms. |
| **14** | **Microalgae** | One of the classifications of eukaryotes, including yeast, mold, and mushrooms. |
| **15** | **Virus** | Infectious particles that parasitize living cells such as plants, animals, and bacteria and can only multiply within cells (e.g. MERS virus) |
| **16** | **Body fluids**(including blood) | Liquids such as blood, lymph, tears, and saliva found in the body of animals or humans |
| **17** | **Extract** | A substance extracted physically or using a solvent (water, organic solvent, supercritical carbon dioxide, etc.) from raw materials of plant, microorganism, animal, water, etc. |
| **18** | **Organic compounds** | A large class of chemical compounds whose molecules contain carbon |
| **19** | **Nucleic acid**(DNA, RNA) | DNA: DNA itself extracted in the form of clone/vector/library such as cDNA/genomicDNA  RNA: RNA itself extracted in the form of clone/vector/library such as microRNA |
| **20** | **Protein**(including derivatives) | Protein: antibody, hormone, enzyme, etc. |
| **21** | **Dry sample** | A material from which moisture has been removed by natural drying, hot air drying, or freeze drying of raw materials of plant, microbial, or animal origin. |
| **22** | **Others** | Frozen Resources-Other, Other Resources with Ambiguous Classification |

**ㅇ** Whether biological resource efficacy data is provided

|  | **Cassification** | **Detailed classification** | **Availability ( ◯ \|▲ \| Blank )** | | | | |
| --- | --- | --- | --- | --- | --- | --- | --- |
|  |  |  | **Mouse** | **Gene** | **Plant** | **Microorganisms** | **Cell** |
| **Efficacy** | **Anti-cancer** | |  |  |  |  |  |
|  | **Anti-bacterial** | **Anti-bacterial** |  |  |  |  |  |
|  |  | **Anti-fungal** |  |  |  |  |  |
|  |  | **Anti-virus** |  |  |  |  |  |
|  | **Anti-inflammatory** | |  |  |  |  |  |
|  | **Anti-aging** | **Anti-aging** |  |  |  |  |  |
|  |  | **Anti-oxidant** |  |  |  |  |  |
|  |  | **Bone health** |  |  |  |  |  |
|  |  | **Eye health** |  |  |  |  |  |
|  |  | **Improve cognitive function** |  |  |  |  |  |
|  | **Insecticide** | **Insecticide** |  |  |  |  |  |
|  | **Anti-diabetic** | **Anti-diabetic** |  |  |  |  |  |
|  |  | **Body sugar control** |  |  |  |  |  |
|  | **Toxicity** | **Toxicity** |  |  |  |  |  |
|  |  | **Cytotoxicity test** |  |  |  |  |  |
|  | **Anti-obesity** | **Anti-obesity** |  |  |  |  |  |
|  | **Cardiovascular health** | **Cardiovascular health** |  |  |  |  |  |
|  |  | **Blood circulation improvement** |  |  |  |  |  |
|  |  | **Blood pressure control** |  |  |  |  |  |
|  | **Respiratory health** | |  |  |  |  |  |
|  | **Digestive Health** | **Stomach health** |  |  |  |  |  |
|  |  | **Viscera health** |  |  |  |  |  |
|  | **Skin health** | **Skin health** |  |  |  |  |  |
|  |  | **Hair health** |  |  |  |  |  |
|  |  | **Whitening** |  |  |  |  |  |
|  | **Immunity** | **Immunity boosting** |  |  |  |  |  |
|  |  | **Immunity suppression** |  |  |  |  |  |
|  | **Environmental improvement** | **Green algae control** |  |  |  |  |  |
|  |  | **Reduction of toxic substances** |  |  |  |  |  |
|  |  | **Specific effect**(particulate matter) |  |  |  |  |  |
|  | **Others** | **Relaxation** |  |  |  |  |  |
|  |  | **Neuro-protection** |  |  |  |  |  |
|  |  | **Liver health** |  |  |  |  |  |
|  |  | **Endoplasmic reticulum stress experiment** |  |  |  |  |  |
|  |  | **Eploration of substances that improve brain disease**(neural cells) |  |  |  |  |  |
