# Supplementary material for: BioOne: a national-scale platform for integrated discovery and utilization of diverse biological resources in South Korea
Source: Genomics Inform. 2026 Apr 8;24:9. doi: 10.1186/s44342-026-00070-x (PMC13130625; doi:10.1186/s44342-026-00070-x)
Supplement: Supplementary file 2 — Supplementary Material 2: Data 2. Benchmarking test results of BioOne. [file 44342_2026_70_MOESM2_ESM.docx]

**[Supplementary Data 2] Benchmarking test results of BioOne.**

*Response time for 30 concurrent users making three requests at 10-second intervals.*

| Retrieval type | Avg. response time (ms) | Min. response time (ms) | Max. response time (ms) | Request processed per second |
| --- | --- | --- | --- | --- |
| Main page | 61 | 54 | 132 | 9.16 |
| Biological resource search | 391 | 326 | 535 | 8.38 |
| Open target platform search | 364 | 306 | 483 | 8.46 |
| Publication search | 362 | 296 | 499 | 8.38 |
| Patent search | 370 | 304 | 1,305 | 8.28 |
| BioOne contents search | 330 | 287 | 440 | 8.39 |
| Cluster biological resource search | 5,716 | 3,097 | 7551 | 3.44 |
