# Supplementary material for: BioOne: a national-scale platform for integrated discovery and utilization of diverse biological resources in South Korea
Source: Genomics Inform. 2026 Apr 8;24:9. doi: 10.1186/s44342-026-00070-x (PMC13130625; doi:10.1186/s44342-026-00070-x)
Supplement: Supplementary file 3 — Supplementary Material 3: Table S1. Classification and functional overview of the 14 national biological resource clusters integrated into BioOne. [file 44342_2026_70_MOESM3_ESM.docx]

**[Supplementary Table S1] Classification and functional overview of the 14 national biological resource clusters integrated into BioOne.**

This table lists the domains of biological materials managed under the Third National Strategy. Each entry includes a brief definition of the resource type and its primary application in research and industry, providing the foundational context for the platform’s cross-domain integration.

| **Clusters** | **Explanation** |
| --- | --- |
| **Animal Models** | Genetically modified or wild-type animals (e.g., mice, zebrafish, Drosophila) used to study human diseases and biological functions. |
| **Cell Lines** | Standardized and quality-controlled immortalized cell lines used for biomedical research and drug testing. |
| **Chemical Compounds** | Synthetic or natural small molecule libraries and chemical entities for drug discovery and biological screening. |
| **Fishery Resources** | Genetic resources and specimens of aquatic organisms, including fish, shellfish, and algae, for aquaculture and marine biotechnology. |
| **Human Brain Resources** | Post-mortem brain tissues, neurological samples, and associated clinical data for neuroscience and brain disorder research. |
| **Human Derived Resources** | Human-derived biospecimens such as blood, DNA, and tissues collected from biobanks for genomic and clinical studies. |
| **Livestock** | Genetic resources of domesticated animals, including embryos, semen, and genomic data for agricultural improvement. |
| **Marine Resources** | Diverse biological resources from marine environments, ranging from microorganisms to macro-invertebrates. |
| **Microbes** | Cultures of bacteria, archaea, fungi, and viruses with industrial, environmental, or academic significance. |
| **Natural Products** | Secondary metabolites and bioactive extracts derived from plants. |
| **Pathogens** | Infectious agents including bacteria, viruses, and fungi that cause diseases in humans. |
| **Seeds** | Plant genetic resources and germplasms preserved for agricultural biodiversity and food security. |
| **Stem cells** | Pluripotent (ESC, iPSC) and multipotent (MSC) stem cells for regenerative medicine and developmental biology research. |
| **Wildlife Resources** | Biological specimens and genetic materials from indigenous wild species for conservation and biodiversity studies. |
| Sources: The Third National Strategy for Management and Utilization of Biological Research Resources(2020). | |
